# Protein Docking Model Evaluation by Graph Neural Networks

**DOI:** 10.3389/fmolb.2021.647915

**Published:** 2021-05-25

**Authors:** Xiao Wang, Sean T. Flannery, Daisuke Kihara

**Affiliations:** ^1^ Department of Computer Science, Purdue University, West Lafayette, IN, United States; ^2^ Department of Biological Sciences, Purdue University, West Lafayette, IN, United States

**Keywords:** protein docking, docking model evaluation, graph neural networks, deep learning, protein structure prediction

## Abstract

Physical interactions of proteins play key functional roles in many important cellular processes. To understand molecular mechanisms of such functions, it is crucial to determine the structure of protein complexes. To complement experimental approaches, which usually take a considerable amount of time and resources, various computational methods have been developed for predicting the structures of protein complexes. In computational modeling, one of the challenges is to identify near-native structures from a large pool of generated models. Here, we developed a deep learning–based approach named Graph Neural Network–based DOcking decoy eValuation scorE (GNN-DOVE). To evaluate a protein docking model, GNN-DOVE extracts the interface area and represents it as a graph. The chemical properties of atoms and the inter-atom distances are used as features of nodes and edges in the graph, respectively. GNN-DOVE was trained, validated, and tested on docking models in the Dockground database and further tested on a combined dataset of Dockground and ZDOCK benchmark as well as a CAPRI scoring dataset. GNN-DOVE performed better than existing methods, including DOVE, which is our previous development that uses a convolutional neural network on voxelized structure models.

## Introduction

Experimentally determined protein structures provide fundamental information about the physicochemical nature of the biological function of protein complexes. With the recent advances in cryo-electron microscopy, the number of experimentally determined protein complex structures has been increasing rapidly. However, experimental methods are costly in terms of money and time. To aid the experimental efforts, computational modeling approaches for protein complex structures, often referred to as protein docking (Aderinwale et al., 2020), have been extensively studied over the past two decades.

Protein docking methods aim to build the overall quaternary structure of a protein complex from the tertiary structure information of individual chains. Similar to other protein structure modeling methods, protein docking can also be divided into two main categories: template-based methods (Tuncbag et al., 2011; Anishchenko et al., 2015), which use a known structure as a scaffold of modeling, and *ab initio* methods, which assemble individual structures and score generated models to choose most plausible ones. In *ab initio* methods, various approaches were used for molecular structure representations (Venkatraman et al., 2009; Pierce et al., 2011). These include docking conformational searches, such as fast Fourier transform (Katchalski-Katzir et al., 1992; Padhorny et al., 2016), geometric hashing (Fischer et al., 1995; Venkatraman et al., 2009), and particle swarm optimization (Moal and Bates, 2010), as well as considering protein flexibility (Gray et al., 2003; Oliwa and Shen, 2015). The development of new methods aims to extend and surpass the capabilities of simple pairwise docking, such as multichain docking (Schneidman‐Duhovny et al., 2005; Esquivel‐Rodríguez et al., 2012; Ritchie and Grudinin, 2016), peptide–protein docking (Kurcinski et al., 2015; Alam et al., 2017; Kurcinski et al., 2020), docking with disordered proteins (Peterson et al., 2017), docking order prediction (Peterson et al., 2018a; Peterson et al., 2018b), and docking for cryo-EM maps (Esquivel-Rodríguez and Kihara, 2012; van Zundert et al., 2015). Researchers have also applied recent advances in deep learning to further boost docking performance (Akbal-Delibas et al., 2016; Degiacomi, 2019; Gainza et al., 2020).

Although substantial improvements have been made in *ab initio* protein docking, selecting near-native (i.e., correct) models out of a large number of produced models, which are often called decoys, is still challenging. The difficulty is partly due to a substantial imbalance in the number of near-native models and incorrect decoys in a generated decoy pool. The accuracy of scoring decoys certainly determines the overall performance of protein docking, and thus, there is active development of scoring functions (Moal et al., 2013) for docking models. Recognizing the importance of scoring, the Critical Assessment of PRediction of Interactions (CAPRI) (Lensink et al., 2018), which is the community-based protein docking prediction experiment, has arranged a specific category of evaluating scoring methods, where participants are asked to select 10 plausible decoys from thousands of decoys provided by the organizers. Over the last two decades, various approaches have been developed for scoring decoys. The main categories include physics-based potentials (Akbal-Delibas et al., 2016; Degiacomi, 2019; Gainza et al., 2020), scoring based on interface shape (Akbal-Delibas et al., 2016; Kingsley et al., 2016; Degiacomi, 2019; Gainza et al., 2020), knowledge-based statistical potentials (Lu et al., 2003; Huang and Zou, 2008), machine learning methods (Fink et al., 2011), evolutionary profiles of interface residues (Nadaradjane et al., 2018), and deep learning methods using interface structures (Wang et al., 2019).

In our previous work, we developed a model selection method for protein docking, that is, DOVE (Wang et al., 2019), which uses a convolutional deep neural network (CNN) as the core of its architecture. DOVE captures atoms and interaction energies of atoms located at the interface of a docking model using a cube of 20^3^ or 40^3^ Å^3^ and judges if the model is correct or incorrect according to the CAPRI criteria (Janin et al., 2003). We showed that DOVE performed better than existing methods. However, DOVE has a critical limitation—since it captures an interface with a fixed-size cube, only a part of the interface is captured when the interface region is too large. This often caused an erroneous prediction. In addition, a 3D grid representation of an interface often includes voxels of void space where no atoms exist inside, which is not efficient in memory usage and may even be detrimental for accurate prediction. In this work, we address this limitation of DOVE by applying a graph neural network (GNN) (Scarselli et al., 2008; Wu et al., 2020), which has previously been successful in representing molecular properties (Duvenaud et al., 2015; Smith et al., 2017; Lim et al., 2019; Zubatyuk et al., 2019). Using a GNN allows all atoms at an interface of any size to be captured in a more flexible manner. The GNN representation of the interface also is rotationally invariant, meaning arbitrary rotations of a candidate model are accounted for when training and predicting docking scores. To the best of our knowledge, this is the first method that applies GNNs to the protein docking problem. Compared to DOVE and other existing methods, GNN-DOVE demonstrated substantial improvement in a benchmark study.

## Materials and Methods

We first introduce the datasets used for training and testing GNN-DOVE. Subsequently, we introduce the graph neural network architecture and the training process of GNN-DOVE.

### Docking Decoy Datasets

To train and test GNN-DOVE, we first used the Dockground dataset 1.0 (available at http://dockground.compbio.ku.edu/downloads/unbound/decoy/decoys1.0.zip) (Liu et al., 2008). Docking decoys in this dataset were built by Gramm-X (Tovchigrechko and Vakser, 2005). The dataset includes 58 target complexes, each with averages of 9.83 correct and 98.5 incorrect decoys. A decoy was considered as correct following the CAPRI criteria (Lensink et al., 2018), which consider interface root mean square deviation (iRMSD), ligand RMSD (lRMSD), and the fraction of native contacts (fnat). The iRMSD is the Cα RMSD of interface residues with respect to the native structure. Interface residues in a complex are defined as all the residues within 10.0 Å from any residues of the other subunit. lRMSD is the Cα RMSD of ligands when receptors are superimposed, and fnat is the fraction of contacting residue pairs, that is, residue pairs with any heavy atom pairs within 5.0 Å, that exist in the native structure.

To remove redundancy, we grouped the 58 complexes using sequence alignment and TM-align (Zhang and Skolnick, 2004). Two complexes were assigned to the same group if at least one pair of proteins from the two complexes had a TM-score of over 0.5 and sequence identity of 30% or higher. This resulted in 29 groups (Table 1). In Table 1, complexes (PDB IDs) of the same group are shown in lower case in a parenthesis followed by the PDB ID of the representative. These groups were split into four subgroups to perform four-fold cross-validation, where three subsets were used for training, while one testing subset was used for testing the accuracy of the model. Thus, by cross-validation, we have four models tested on four independent testing sets. Among the training set, we used 80% of the complexes (i.e., unique dimers) for training a model and the remaining 20% of the complexes as a validation set, which was used to determine the best hyper-parameter set for training. In the results, the accuracy of targets when treated in the testing set was reported. To have a fair comparison with DOVE (Wang et al., 2019), DOVE was also newly trained and tested using this protocol.

**TABLE 1:** Dockground dataset splits for training and testing GNN.

Fold	PDB ID
1	1A2K, 1E96 (1he1, 1he8, 1wq1), 1F6M, 1MA9 (2btf), 1G20, 1KU6, 1T6G, 1UGH, 1YVB, 2CKH, 3PRO
2	1AKJ (1p7q, 2bnq), 1DFJ, 1NBF (1r4m, 1xd3, 2bkr), 1GPW, 1HXY, 1U7F, 1UEX, 1ZY8, 2GOO, 1EWY
3	1AVW (1*b*th, 1bui, 1cho, 1ezu, 1ook, 1oph, 1ppf, 1tx6, 1xx9, 2fi4, 2kai, 1r0r, 2sni, 3sic)
4	1BVN (1tmq), 1F51, 1FM9, 1A2Y (1g6v, 1gpq, 1jps, 1wej, 1l9b, 1s6v), 1W1I, 2A5T, 3FAP

There are in total 29 representative targets shown in the upper case; targets in the lower case in a parenthesis indicate that they belong to the same group.

Subsequently, we further trained and validated the GNN-DOVE network with a combined dataset of Dockground (ver 1.0) and ZDOCK (ver 4.0) (Hwang et al., 2010), which includes 58 target complexes from Dockground and 120 target complexes from ZDOCK. ZDOCK has 110 more targets, but they were discarded because either GOAP (Zhou and Skolnick, 2011) or ITScore (Huang and Zou, 2008) failed to process them, or fnat could not be computed due to inconsistency of the sequence in the structures provided in the ZDOCK dataset from the native complex structure in PDB. The same criteria mentioned above were used to group the targets into 71 groups. Among them, we used 45 groups for training, 11 groups for validation, and 15 groups (19 complexes) for testing. Since a decoy set for each target in ZDOCK is much larger (around 54,000) than Dockground, we reduced the number of ZDOCK decoys for a target to 400. Up to 200 correct decoys (i.e., decoys with an acceptable or higher CAPRI quality) were selected if available, including at most 50 high-quality decoys, at most 50 medium-quality decoys, and the rest were selected from acceptable quality decoys. Then, the remaining 400 decoys were filled with negative decoys. One-third of negative decoys were selected from those with an iRMSD less than 7 Å, another third came from those with an iRMSD between 7 and 10 Å, and the rest came from those with ones with an iRMSD over 10 Å.

Finally, we tested GNN-DOVE on decoy sets of 13 targets in the CAPRI Score_set (Lensink and Wodak, 2014), which consists of 13 scoring targets from the CAPRI round 13 to round 26 (Janin, 2010; Janin, 2013). Each decoy set included 500 to 2,000 models generated using different methods by CAPRI participants.

### The GNN-DOVE Algorithm

**FIGURE 1:**
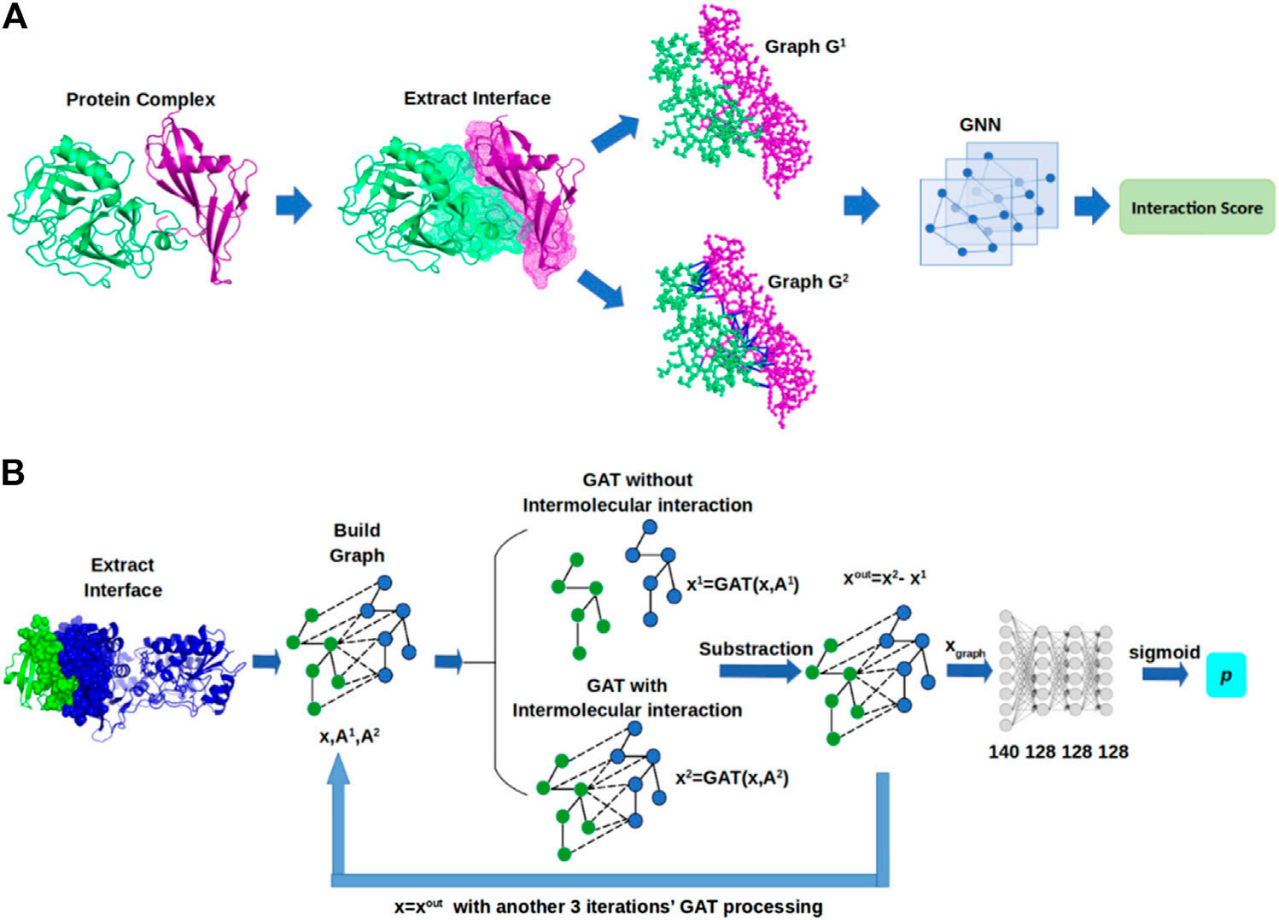
Framework of GNN-DOVE. GNN-DOVE extracts the interface region of protein complex and further reconstructs graph with/without intermolecular interactions as input, then outputs the probability that indicates if the input structure is acceptable or not. **(A)** Overall logical steps of the pipeline. **(B)** Architecture of the GNN network with the gated graph attention mechanism.

In this section, we describe GNN-DOVE, which uses the graph neural network. The GNN-DOVE algorithm is inspired by a recent work in drug–target interactions (Lim et al., 2019), which designed a two-graph representation for capturing intermolecular interactions for protein–ligand interactions. We will first explain how the 3D structural information of a protein–complex interface is embedded as a graph. Then, we describe how we used a graph attention mechanism to focus on the intermolecular interaction between a receptor and a ligand protein. The overall protocol is illustrated in Figure 1. For an input protein docking decoy, the interface region is identified as a set of residues located within 10.0 Å of any residues of the other protein. A residue–residue distance is defined as the shortest distance among any heavy atom pairs across the two residues. Using the extracted interface region, two graphs are built representing two types of interactions: the graph $G^{1}\;$ describes heavy atoms at the interface region, which only considers the covalent bonds between atoms of interface residues within each subunit as edges. Another graph $G^{2}$ connects both covalent (thus includes $G^{1}\;$) and non-covalent residue interaction as edges, where a non-covalent atom pair is defined as those which are closer than 10.0 Å of each other. Both graphs will be processed by a graph neural network (GNN) to output a score, which is a probability that the docking decoy has a CAPRI acceptable quality (thus making higher scores better).

### Building Graphs

A key feature of this work is the graph representation of an interface region of a complex model. Graph *G* is defined by *G =* (*V*, *E*, and *A*), where *V* denotes the node set, *E* is a set of edges, and *A* is the adjacency matrix, which numerically represents the connectivity of the graph. For a graph *G* with *N* nodes, the adjacency matrix *A* has a dimension of N*N, where $A_{ij}>0$ if the *i-*th node and the *j-*th node are connected, and $A_{ij}=0$ otherwise. The adjacency matrix $A^{1}$ for graph $G^{1}$ describes covalent bonds at the interface and thus defined as follows:

$$A_{ij}^{1}=\{\begin{array}{c} 1\;\;if\;atom\;i\;and\;atom\;j\;are\;connected\;by\;a\;\mathrm{cov}alent\;bond\;or\;if\;i=j\\ 0\;\;otherwise \end{array}.$$ (1)

The matrix $A^{2}$ for $G^{2}$ describes both covalent bonds and non-covalent interactions between atoms within 10.0 Å to each other. It is defined as follows:

$$A_{ij}^{2}=\{\begin{array}{c} A_{ij}^{1},\;\;\;if\;i,j\in receptor\;or\;i,j\in ligand\;\\ e^{\frac{-{\left(d_{ij}-\mu \right)}^{2}}{\sigma }},\;\;if\;d_{ij}\leq 10\;\;Å\;and\;i\in receptor\;\;and\;j\in ligand;\\ or\;if\;d_{ij}\leq 10\;\;Å\;and\;j\in receptor\;\;and\;i\in ligand\\ 0,\;\;otherwise \end{array}$$ (2)

where $d_{ij}$ denotes the distance between the *i-*th and the *j-*th atoms. μ and σ are learnable parameters, whose initial values are 0.0 and 1.0, respectively. The formula $e^{-{\left(d_{ij}-\mu \right)}^{2}/\sigma }$ decays as the distance increases between atoms.

Compared to the previous voxel representation used in DOVE, the graph representation encodes the distance information more flexibly and naturally. Note that the representation is rotationally invariant and any size of interaction regions can be taken into analysis. Also, memory usage is more efficient as void spaces are not represented as is needed for the voxel representation.

As for the node features in the graph, we considered the physicochemical properties of atoms. We used the same features as used in previous works (Lim et al., 2019; Torng and Altman, 2019) as shown in Table 2. Thus, the length of a feature vector of a node from Table 2 was 23 (=5 + 6+5 + 6+1), which was embedded by a one-layer fully connected (FC) network into 140 features.

**TABLE 2:** Atom features.

Features	Representation
Atom type	C, N, O, S, H (one hot)
The degree (connections) of atom	0, 1, 2, 3, 4, 5 (one hot)
The number of connected hydrogen atoms	0, 1, 2, 3, 4 (one hot)
The number of implicit valence electrons	0, 1, 2, 3, 4, 5 (one hot)
Aromatic	0 or 1

### Attention and Gate-Augmented Mechanism

The constructed graphs are used as the input to the GNN. More formally, graphs are the adjacency matrix A^1^ and A^2^, and the node features, $x^{in}=\left\{x_{1}^{in},x_{2}^{in},\;\cdots ,x_{N}^{in}\right\}$ with $x\in {\mathbb{R}}^{F}$, where F is the dimension of the node feature.

We first explain the attention mechanism of our GNN. With the input graph of $x^{in}$, the pure graph attention coefficient is defined in Eq. 3, which denotes the relative importance between the *i-*th and the *j-*th node:

$$e_{ij}=x_{i}^{\prime Τ}Ex_{j}^{\prime }+x_{j}^{\prime Τ}Ex_{i}^{\prime },$$ (3)

where $x_{i}^{\prime }$ and $x_{j}^{\prime }$ are the transformed feature representations defined by $x_{i}^{\prime }=Wx_{i}^{in}$ and $x_{j}^{\prime }=Wx_{j}^{in}$. $W,E\in {\mathbb{R}}^{F\times F}$ are learnable matrices in the GNN. $e_{ij}$ and $e_{ji}$ become identical to satisfy the symmetrical property of the graph by adding $x_{i}^{\prime Τ}Ex_{j}^{\prime Τ}$ and $x_{i}^{\prime Τ}Ex_{i}^{\prime }$. The coefficient will only be computed for *i* and *j* where $A_{ij}>0$.

Attention coefficients will also be computed for elements in the adjacency matrices. They are formulated in the following form for the element (*i*, *j*):

$$a_{ij}=\frac{\exp\left(e_{ij}\right)}{\sum_{j\in N_{i}}\exp\left(e_{ij}\right)}A_{ij},$$ (4)

where $a_{ij}$ is the normalized attention coefficient for the *i-*th and the *j-*th node pair, $e_{ij}$ is the symmetrical graph attention coefficient computed in Eq. 3, and $N_{i}$ is the set of neighbors of the *i-*th node that includes interacting nodes *j* where $A_{ij}>0$. The purpose of Eq. 4 is to consider both the physical structure of the interaction, *A*_*ij*_, and the normalized attention coefficient, e_ij_, to define the attention.

Based on the attention mechanism, the new node feature of each node is updated by considering its neighboring nodes, which is a linear combination of the neighboring node features with the final attention coefficient $a_{ij}$:

$$x_{i}^{\prime \prime }=\sum_{j\in N_{i}}a_{ij}x_{j}^{\prime }.$$ (5)

Furthermore, the gate mechanism is further applied to update the node feature since it is known to significantly boost the performance of GNN (Zhang et al., 2018). The basic idea is similar to that of ResNet (He et al., 2016), where the residual connection from the input helps to avoid information loss, alleviating the gradient collapse problem of the conventional backpropagation. The gated graph attention can be viewed as a linear combination of $x_{i}$ and $x_{i}^{\prime \prime }$, as defined in Eq. 6:

$$x_{i}^{out}=c_{i}x_{i}+\left(1-c_{i}\right)x_{i}^{\prime \prime },$$ (6)

where $c_{i}=\sigma \left[D\left(x_{i}\left|\right|x_{i}^{\prime \prime }\right)+b\right]$, $D\in {\mathbb{R}}^{2F}\;$ is a weight vector that is multiplied (dot product) with the vector $x_{i}\left|\right|x_{i}^{\prime \prime }$, and b is a constant value. Both D and b are learnable parameters and are shared among different nodes. $x_{i}\left|\right|x_{i}^{\prime \prime }$ denotes the concatenation vector of $x_{i}\;and\;x_{i}^{\prime \prime }$.

We refer to attention and gate-augmented mechanism as the gate-augmented graph attention layer (GAT). Then, we can simply denote $x_{i}^{out}=GAT\left(x_{i}^{in},A\right)$. The node embedding can be iteratively updated by GAT, which aggregates information from neighboring nodes.

### Graph Neural Network Architecture of GNN-DOVE

Using the GAT mechanism described before, we adopted four layers of GAT in GNN-DOVE to process the node embedding information from neighbors and to output the updated node embedding (Figure 1B). For the two adjacency matrices $A^{1}\;$ and $A^{2}$, we used a shared GAT. The initial input of the network is atom features. With two matrices, $A^{1}\;$ and $A^{2}$, we have $x_{1}=GAT\left(x^{in},A^{1}\right)$ and $x_{2}=GAT\left(x^{in},A^{2}\right)$. To focus only on the intermolecular interactions within an input protein complex model, we subtracted the embedding of the two graphs as the final node embedding. By subtracting the updated embedding $x_{1}$ from $x_{2}$, we can capture the aggregation information that only comes from the intermolecular interactions with other nodes in the protein complex model. Thus, the output node feature is defined as

$$x^{out}=x^{2}-x^{1}.$$ (7)

Then, the updated $x^{out}$ will become $x^{in}$ to iteratively augment the information through the three following GAT layers. After the node embeddings were updated by the four GAT layers, the node embedding of the whole graph was summed up as the entire graph representation, which is considered as the overall intermolecular interaction representation of the protein complex model:

$$x_{graph}=\sum_{k\in G}x_{k}.$$ (8)

Finally, FC layers were applied to $x_{graph}$ to classify whether the protein complex model is correct or incorrect. In total, four FC layers were applied. The first layer takes 140 feature values from Eq. 8. The three subsequent layers have a dimension of 128. RELU activation functions were used between the FC layers, and a sigmoid function was applied for the last layer to output a probability value.

The source code of GNN-DOVE is available at https://github.com/kiharalab/GNN_DOVE.

### Training Networks

Since the dataset was highly imbalanced with more incorrect decoys than acceptable ones, we balanced the training data by sampling the same number of acceptable and incorrect decoys in each batch. We sampled the same number of correct and incorrect decoys. To achieve this, a positive (i.e., correct) decoy may be sampled multiple times in one epoch of training.

For training, cross-entropy loss (Goodfellow et al., 2016) was used as the loss function, and the Adam optimizer (Kingma and Ba, 2015) was used for parameter optimization. To avoid overfitting, a dropout (Srivastava et al., 2014) of 0.3 was applied for every layer, except the last FC layer. Models were trained for 100 epochs with a batch size of 32. Weights of every layer were initialized using the Glorot uniform (Glorot and Bengio, 2010) to have a zero-centered Gaussian distribution, and bias was initialized to 0 for all layers.

First, we performed four-fold cross-validation on the Dockground dataset (Table 1). For fold 1, where we used the fold 1 subset as testing and the other three subsets for training and validation, 16 hyper-parameter combinations with learning rates of 0.2, 0.02, 0.002, and 0.0002 and a weight decay in Adam of 0, 1e-1, 1e-2, 1e-3, 1e-4, and 1e-5 were tested. Among these combinations, we found a learning rate of 0.002 with a weight decay of 0 achieved the highest accuracy on the validation set. We used this parameter combination throughout the other three folds in the cross-validation. The training process generally converged after approximately 30 epochs.

Next, we used the combined dataset of Dockground and ZDOCK for further training. We adopted transfer learning on this dataset by starting from the models pretrained on the Dockground dataset. The training was performed in two stages: In the first stage, nine hyper-parameter combinations with learning rates of 0.002, 0.0002, and 0.00002 and weight decay of 1e-4, 1e-5, and 0 were tested on the fold 1 model. We found that a combination of a learning rate of 0.0002 and weight decay of 0 performed the best when evaluated on its validation set. We used this hyper-parameter combination to train the fold 2, 3, and 4 models and selected the fold 1 model for further training because it showed the highest accuracy on the validation set. In the second stage, we used a smaller learning rate of 0.00002 and weight decay 0 to further fine tune the fold 1 model for another 30 epochs. The resulting model was evaluated on the testing set of the combined Dockground and ZDOCK dataset. Further, we applied the model to the dataset of CAPRI scoring targets.

### DOVE

We compared the performance of GNN-DOVE with its predecessor, DOVE. Here, we briefly describe the DOVE algorithm. DOVE is a CNN-based method for evaluating protein docking models. It first extracts the interface region of an input protein complex model, and the region is put into a 40*40*40 Å^3^ cube as input. A seven-layer CNN, which consists of three convolutional layers, two pooling layers, and two fully connected layers, was adopted to process the voxel input. The output of DOVE is the probability that indicates whether the input model is acceptable or not. For input features, DOVE took atom types as well as atom-based interaction energy values from GOAP (Zhou and Skolnick, 2011) and ITScore (Huang and Zou, 2008). Since voxelized structure input is not rotationally invariant, DOVE needed to augment training data by rotations.

## Results

### Performance on the Dockground Dataset

We evaluated the performance of GNN-DOVE on the Dockground dataset. GNN-DOVE was compared with DOVE and five other existing structure model scoring methods, such as GOAP (Zhou and Skolnick, 2011), ITScore (Huang and Zou, 2008), ZRANK (Pierce and Weng, 2007), ZRANK2 (Pierce and Weng, 2008), and IRAD (Vreven et al., 2011). The test set results were reported for GNN-DOVE and DOVE. Both GOAP and ITScore were run in two different ways. First, as originally designed, the entire complex structure model was input. The other way was to input only the interface residues that are within 10 Å of the interacting protein (denoted as GOAP-Interface and ITScore-Interface). Thus, GNN-DOVE was compared with a total of eight methods. As for DOVE, we used a cube size of 40^3^ Å^3^ and heavy atom distributions as input feature because this setting performed the best among other settings tested on the Dockground dataset in the original paper (Wang et al., 2020) (Figure 4 in the paper, the setting was named as DOVE-Atom 40). For this work, DOVE was newly retrained using the same four-fold cross-validation as GNN-DOVE.

**FIGURE 2:**
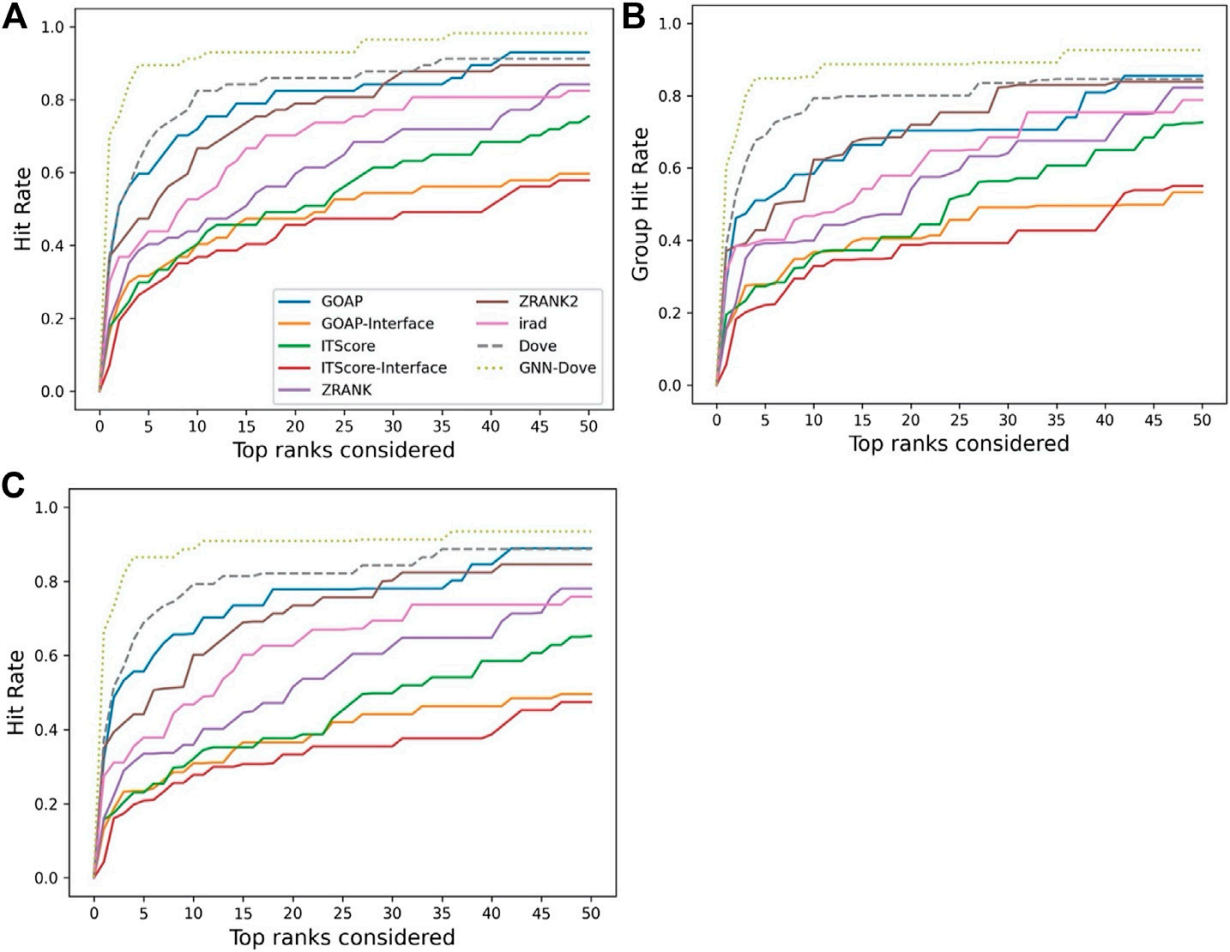
Performance on the Dockground dataset. GNN-DOVE was compared with DOVE and seven other scoring methods. **(A)** The panel shows the fraction of target complexes among the 58 complexes in the benchmark set for which a method selected at least one acceptable model (within top *x* scored models). **(B)** Considering the complexes are grouped into 29 groups, we also compared the hit rate of different methods based on the group classification. The hit rates for complexes in each group were averaged and then re-averaged over the 29 groups. **(C)** Results when 46 complex groups were considered that were formed with interface similarity. The hit rates for complexes in each group were averaged and then re-averaged over the 46 groups.

Figure 2 shows the hit rate of GNN-DOVE in comparison with the other methods. A hit rate of a method is the fraction of target complexes where the method ranked at least one acceptable model based on the CAPRI criteria within each top rank. Targets were evaluated when they were in the heldout testing set from the four-fold cross-testing we performed. In Figure 2, we show three panels. Panel A shows the fraction of targets where a method had at least one hit among each rank cutoff. Panel B shows the hit rates for a method were averaged first within each of the 29 groups, and then re-averaged over the groups. Panel C shows the hit rate when targets with similar interface structures were grouped.

Figure 2 shows that GNN-DOVE (dotted line in light green) performed better than the other methods. GNN-DOVE was able to rank correct models within earlier ranks in many target complexes. Within the top 10 rank, GNN-DOVE achieved a hit rate of 89.7%, while the next best method, DOVE, achieved 81.0%, and the third best method, GOAP, obtained 70.7% (Figure 2A). When we further compared the hit rates considering the target groups (Figure 2B), GNN-DOVE consistently outperformed other methods. The gap between GNN-DOVE and DOVE against the other existing methods also increased. Among the other seven existing methods, GOAP showed the highest hit rate at 5th rank, followed by ZRANK2 in both panels, while ITScore-Interface had the lowest hit rates on this dataset. In Figure 2C, we evaluated the methods’ performance when target complexes were grouped considering their docking interface area similarity, which was evaluated by TM-Score. For a complex, an interface was defined as residues that are closer than 10 Å to any residue of the docking partner. To run TM-align to obtain TM-Score for two interfaces, we prepared two versions of PDB files for each interface: one with residues from the receptor first followed by residues from the ligand and the other with the opposite order. Then, we computed TM-Score for four combinations of the files from the two interfaces and selected the largest TM-Score among them. A pair of interfaces was grouped if one of the computed TM-score values of the interface regions was 0.5 or higher. This process formed 46 groups. The hit rate was computed for each complex first, then averaged within each group, and finally re-averaged across 46 groups. GNN-DOVE still showed the highest hit rate among the methods compared when considering top 10 ranks.

**FIGURE 3:**
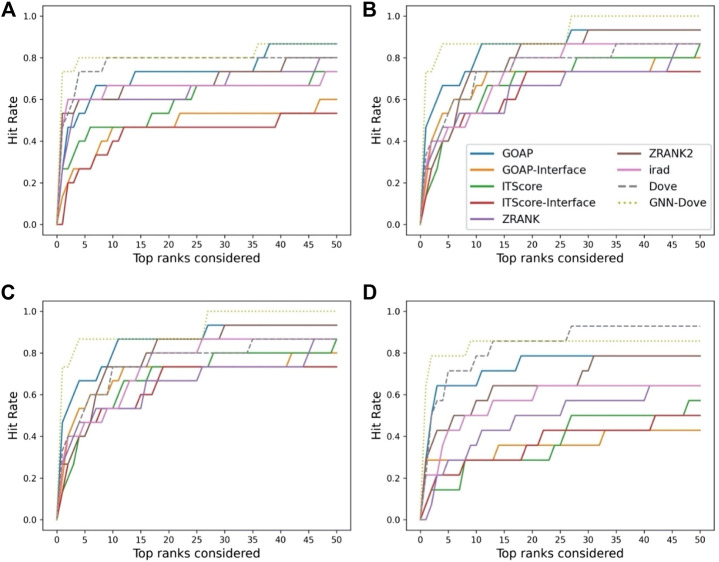
The hit rate is shown for each fold in the cross validation on the Dockground dataset. Protein complexes in the test set of each fold are listed in Table 1. In the same way as Figure 2A, a hit rate was computed for individual complexes separately and averaged over the complexes. **(A)** The hit rate of the fold 1 test set. The model was trained on the fold 2, 3, and 4 subsets. **(B)** The fold 2 test set. **(C)** The fold 3 test set. **(D)** The fold 4 test set.

In Figure 3, we show results on each test set from the four-fold cross validation. GNN-DOVE showed the highest hit rate in early ranks.

**FIGURE 4:**
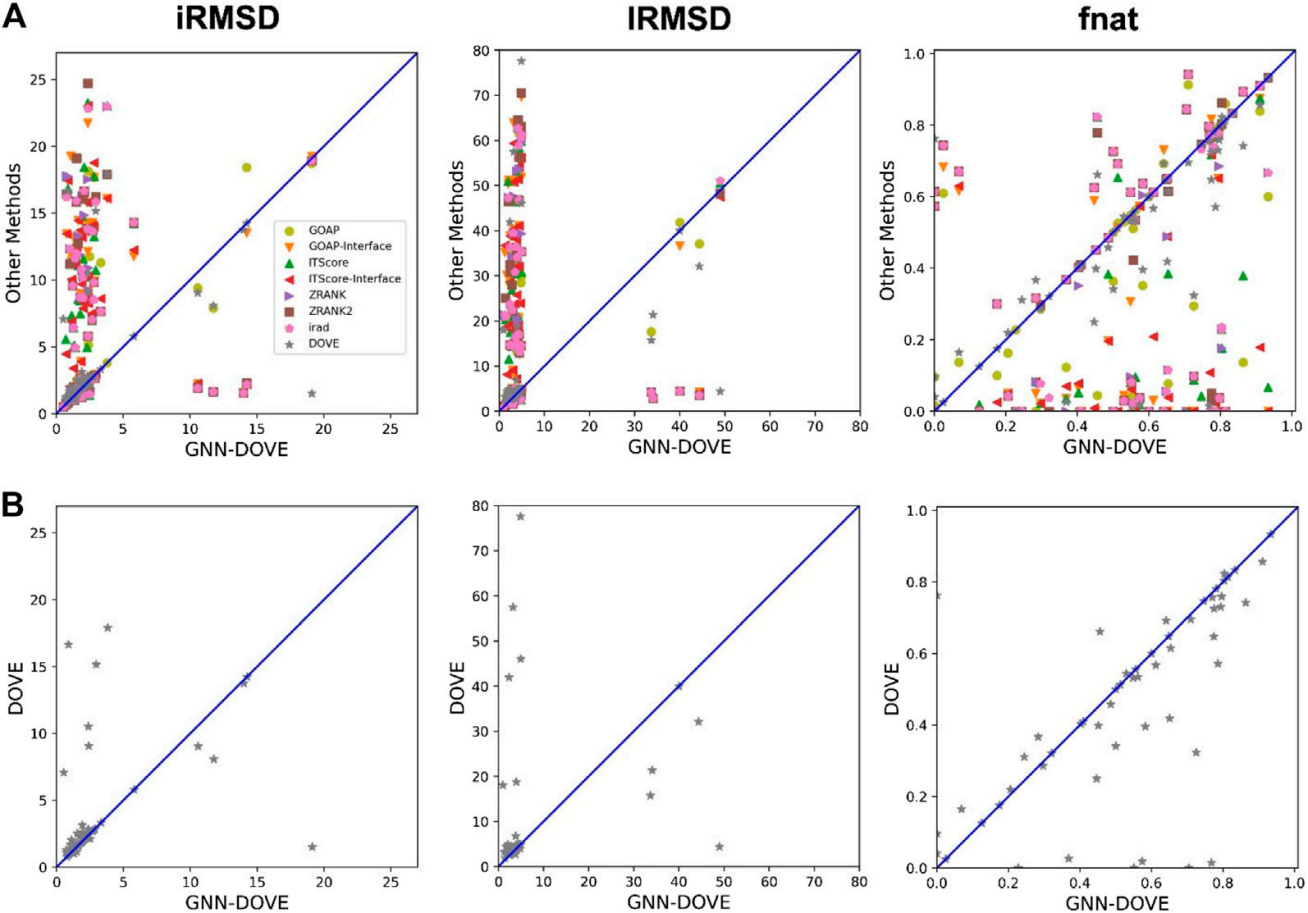
Comparison of iRMSD, lRMSD, and fnat. For each method, the best value among the top 10 scored decoys was plotted. **(A)** Comparison against all eight methods. **(B)** Comparison against DOVE.

In Figure 4, we compared iRMSD, lRMSD, and fnat values of the methods. These metrics are used for defining the quality levels in CAPRI. The best value among the top 10 ranked decoys was plotted. For the majority of the cases (49 out of the 58 targets), GNN-DOVE selected a decoy within an iRMSD of 4 Å (one of the criteria for the acceptable quality level in CAPRI). This is in sharp contrast to the other methods (Figure 4A), where the iRMSD of many targets they selected were larger (worse) than GNN-DOVE. In terms of iRMSD, the second best method was DOVE, where 44 targets were within an iRMSD of 4 Å. A similar situation was observed for lRMSD. GNN-DOVE selected a decoy within an lRMSD of 10 Å (one of the criteria for the acceptable quality level in CAPRI) for 50 targets, while the second best method, DOVE, selected 45 targets within 10 Å lRMSD. In terms of fnat (larger being more accurate), GNN-DOVE only missed 5 targets in selecting at least one model with an fnat over 0.1 (one of the criteria for acceptable quality level in CAPRI). The plot shows that GNN-DOVE had a larger fnat value than the other existing methods for most of the targets, as indicated by many data points below the diagonal line.

Figure 4B compares GNN-DOVE against DOVE. In terms of iRMSD, lRMSD, and fnat, GNN-DOVE outperformed DOVE for 26 targets (22 ties), 27 targets (20 ties), and 27 targets (17 ties targets), respectively. Overall, GNN-DOVE outperformed the eight existing methods for all three metrics.

### T-SNE Analysis

**FIGURE 5:**
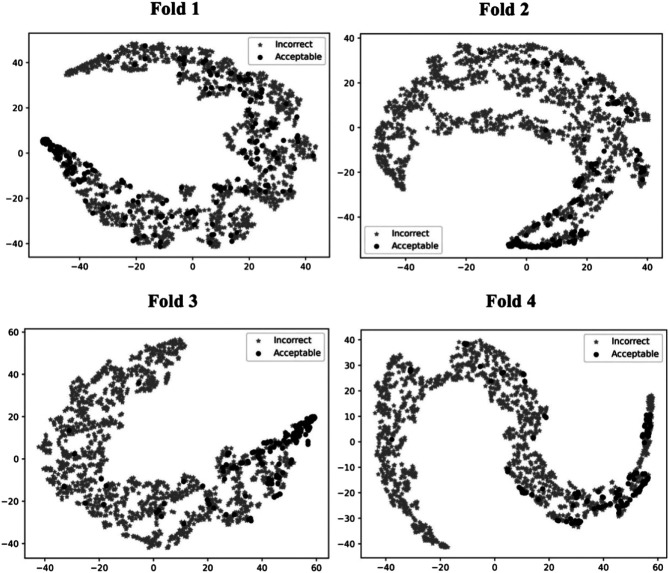
t-SNE plots of decoy selection. Decoys from all the testing target complexes in the four different folds in the cross-testing are plotted, which in total include 580 correct decoys (black circles) and 5,591 incorrect decoys (gray stars). Encoded features of those decoys are taken from the output of the last fully connected layer of GNN, which is a vector of 128 elements. To visualize the different embedding, we use t-SNE to project them into a 2D space. The four panels correspond to the embedding of models on the four-fold testing sets.

To illustrate how GNN-DOVE classified decoys, we used t-SNE (Maaten and Hinton, 2008) to visualize GNN-DOVE’s encoding of decoys in Figure 5. t-SNE is a dimension-reduction method to visualize similarities of high-dimensional data points. Since we employed a four-fold cross-validation, a plot was provided for each of the four testing sets. In all the plots, particularly in Fold 3 and Fold 4, most of the acceptable decoys (black circles) were distinguished from incorrect ones (gray crosses), which indicates a good representation and generalization ability of the graph neural networks for this problem.

### Examples of Decoys for Comparison With DOVE

**FIGURE 6:**
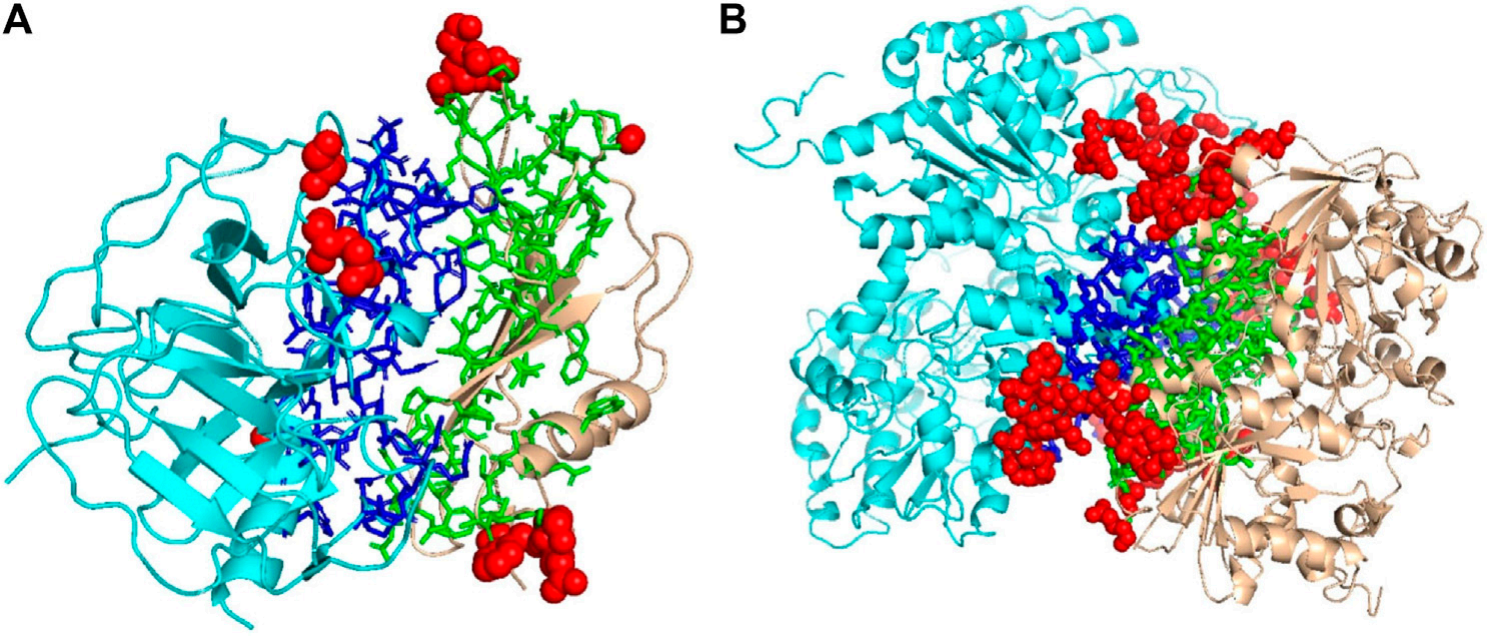
Examples of decoys with an acceptable quality but not selected within the top 10 by DOVE. Two subunits docked are shown in cyan and light brown, and the interface regions of the two subunits are presented in the stick representation and in blue and green, respectively. To highlight the missed atoms from the input cube of DOVE, they are shown in red spheres. **(A)** A medium-quality decoy for 1bui. iRMSD: 2.54 Å, lRMSD: 2.93 Å, fnat: 0.551. **(B)** A medium-quality decoy for 1g20. iRMSD: 2.14 Å, lRMSD: 3.86 Å, fnat: 0.453.

We mentioned above that a limitation of DOVE is its usage of a fix-sized cube of 40^3^ Å^3^, which cannot capture the entire interface region if the interface is too large to fit in the cube. Here, we show two examples of such cases, which led to misclassification by DOVE but correct classification by GNN-DOVE. In Figure 6, the interface region of a decoy is shown in blue and green, and the atoms that did not fit in the cube are shown in a sphere representation in red.

The first example (Figure 6A) shows a decoy of a protein complex of plasminogen and staphylokinase (PDB ID: 1bui), which has an acceptable quality by the CAPRI criteria. For this decoy, 59 atoms (in red) out of 1,022 atoms at the interface were not included in the cube. Because of this, it was ranked the 65th out of 110 decoys by DOVE, while it was ranked 15th by GNN-DOVE. For this target, GNN-DOVE ranked five hits within the top 10 scoring decoys and eight hits within the top 20. In contrast, DOVE could not rank any hit within the top 20. The first hit by DOVE was found at the 35th rank.

The second example (Figure 6B) is an acceptable model for the nitrogenase complex (PDB ID: 1g20). As shown, many interface atoms, 497 out of 1,843, were outside the cube. DOVE ranked this decoy 28th, while GNN-DOVE ranked this decoy 10th. DOVE had 0 hits within the top 10 and had only one hit within top 20. On the other hand, GNN-DOVE was very successful for this target, where all the top 10 selections were correct models.

### Performance on the Combined Dockground and ZDOCK Dataset

Next, we examined the performance of GNN-DOVE on the 19 complexes in the test set of the combined Dockground and ZDOCK dataset. In Table 3, we showed the total number of hits among top 10 ranks by GNN-DOVE and the same five other methods, that is, GOAP, ITScore, ZRANK, ZRANK2, and IRAD, as we used in Figures 2–4. GNN-DOVE achieved the highest hit rate of 0.842, followed by ZRANK with 0.789. GNN-DOVE ranked at the top among the methods consistently when the group hit rate was considered. We note that some of the existing methods performed perfectly for specific complexes, choosing 10 hits within the top 10. However, many methods failed to select any top hits for other target complexes. In contrast, GNN-DOVE showed the most stable performance across different complexes.

**TABLE 3:** Performance on the Dockground+ZDOCK testing dataset.

ID	GNN-DOVE	GOAP	ITScore	ZRANK	ZRANK2	IRAD	Total
1AK4	1	10	1	1	7	0	179
1AY7	8	0	3	9	8	8	176
1EER	0	0	0	0	3	0	41
1GLA	5	1	0	8	4	8	165
1HCF	9	0	8	3	3	7	183
1JIW	3	0	2	0	1	2	106
1JTG	8	0	10	10	0	10	177
1KAC	7	0	5	8	2	6	183
1KTZ	0	1	0	1	3	0	77
1MAH	9	0	8	9	0	9	179
2MTA	7	0	4	9	0	9	186
2VDB	9	1	9	7	2	6	173
3D5S	7	0	10	6	1	5	156
1BUH (1)	3	8	9	6	4	9	183
1FQ1 (1)	0	0	0	0	0	0	20
1JWH (1)	6	6	7	6	2	8	171
2OZA (1)	1	0	1	0	0	0	19
1EFN (2)	1	0	0	4	3	4	130
1GCQ (2)	2	9	0	1	8	4	142
Hit rate	0.842	0.368	0.684	0.789	0.737	0.737	—
Group HR	0.867	0.333	0.717	0.833	0.767	0.767	

In the ID column, the number in a parentheses indicates which group the target belongs to. Thus, four complexes belong to the same similarity group, and the other two belong to another group. The rest of the complexes are single entry groups. Group HR indicates the group hit rate. In Group HR, the fraction of complexes within each group that have at least one hit (acceptable model) within the top 10 ranks was first computed, and then averaged across all the groups. The total column indicates the total number of acceptable docking models for a given target.

### Performance on the CAPRI Scoring Dataset

Finally, we evaluate GNN-DOVE on another independent dataset, the CAPRI Score_set. This dataset was chosen to be able to compare GNN-DOVE on a larger number of existing methods which participated in the corresponding CAPRI rounds. In Table 4, we show detailed results of GNN-DOVE and the other five methods for each target. For each method, the number of decoys within the quality categories of acceptable, medium, and high (in this order) of the top 10 models are listed.

**TABLE 4:** Performance on the CAPRI scoring dataset.

ID	GNN-DOVE	GOAP	ITScore	ZRANK	ZRANK2	IRAD	Total
(T29)	2/0/0	1/0/0	0/0/0	0/0/0	2/2/0	1/1/0	167/78/2
(T30)	1/0/0	0/0/0	0/0/0	0/0/0	0/0/0	0/0/0	2/0/0
T32	0/0/0	1/0/0	0/0/0	0/0/0	0/0/0	0/0/0	15/3/0
T35	1/0/0	0/0/0	0/0/0	0/0/0	0/0/0	0/0/0	3/0/0
(T37)	0/0/0	1/0/0	3/0/1	1/0/0	4/1/0	4/1/0	99/46/11
T39	0/0/0	0/0/0	0/0/0	0/0/0	0/0/0	0/0/0	4/3/0
(T40)	4/4/0	1/0/1	7/3/4	1/1/0	9/8/1	3/3/0	588/206/193
T41	5/0/0	4/2/2	1/1/0	4/0/0	2/0/0	3/0/0	371/120/2
T46	1/0/0	0/0/0	0/0/0	5/0/0	6/0/0	6/0/0	24/0/0
T47	9/4/5	10/0/10	2/1/0	9/5/4	9/3/5	10/2/7	611/307/278
T50	6/0/0	0/0/0	4/1/0	0/0/0	2/0/0	2/0/0	133/36/0
T53	2/2/0	7/6/0	3/0/0	1/0/0	7/3/0	4/2/0	130/17/0
(T54)	0/0/0	0/0/0	0/0/0	0/0/0	0/0/0	0/0/0	19/1/0
Hit	9/3/1	7/2/3	6/4/2	6/2/1	8/5/2	8/5/1	13/10/5
Hit-NR	6/2/1	4/2/2	4/3/0	4/1/1	5/2/1	5/2/1	8/7/2

The IDs in parentheses are those which have structure or sequence similarity to one of the complexes used in training. Results for a complex by a method have three numbers separated by /. The first number is the number of decoys selected within the top 10 ranked models, which has an acceptable or better quality. The second and third numbers are the number of models with medium or higher quality, and the number of high-quality models. The numbers in the total column indicate the total number of decoys in the three quality classifications in the decoy set of each target. The last two rows report the summary of the performance. Three numbers are the number of targets where the method identified at least one acceptable or higher-quality models, at least one medium- or higher-quality models, or at least one high-quality model, respectively. The hit row lists the results when all 13 targets were considered. Hit-NR only considers targets that are not in parentheses.

GNN-DOVE had hits for the largest number of targets, that is, nine, when decoys of acceptable or higher quality were considered. When decoys in a medium or higher quality were considered, ITScore, ZRANK2, and IRAD had hits for five targets, while GNN-DOVE had hits for three targets. It is worth noting that GNN-DOVE successfully identified correct models in two difficult targets, T30 and T35, which only contained two and three acceptable models in the decoy sets, while all the other methods failed to select any correct decoys among the top 10.

In Table 5, we further compared GNN-DOVE with the top groups who participated in the model scoring task for the 13 CAPRI scoring targets. The results were taken from Table 2 of the article by Geng et al. (2020). In total, 37 scoring groups have submitted their scores during this challenge and among them we list here only groups with five or more submitted targets. In addition to the CAPRI participants the table also includes the latest protein docking evaluation approaches, iScore (Geng et al., 2020) and GraphRank (Geng et al., 2020).

**TABLE 5:** Ranking of GNN-DOVE among other scorer groups on the CAPRI scoring dataset.

Group	Performance		# Submitted targets
	All	Nonredundant	
iScore	9/6/2	6/5/1	13 (8)
GNN-DOVE	9/3/1	6/2/1	13 (8)
GraphRank	8/4/1	5/3/1	13 (8)
Bates	8/4/1	5/2/0	10 (5)
Bonvin	8/3/2	5/2/1	9 (5)
Weng	8/2/3	5/2/1	9 (6)
Zou	7/1/4	5/1/2	9 (6)
Wang	6/3/2	4/2/1	6 (4)
Fernandez-Recio	5/3/2	4/4/1	8 (7)
Elber	5/1/1	4/1/0	5 (4)
Wolfson	4/0/1	1/0/0	5 (2)
Camacho	3/1/2	1/1/1	5 (2)

Results of the existing methods were taken from Table 2 of the article by Geng et al. (2020). The numbers in the nonredundant column only considered targets in Table 4 that are not in the parentheses. The last column shows the number of targets that each group has submitted their prediction among the 13 targets listed in Table 4. The numbers in parentheses report the number of submitted targets among those which do not have similarity to the training set we used (i.e., discarding the targets in parentheses in Table 4).

GNN-DOVE tied with iScore when decoys of acceptable or higher quality were considered. When medium- or higher-quality decoys were considered, GNN-DOVE performed second to iScore. In this list, except for GNN-DOVE, iScore, and GraphRank, all the other groups were human groups, which may have used manual intervention using expert knowledge. Thus, the results show that GNN-DOVE is also highly competitive against human experts.

## Discussion

In this work, we developed GNN-DOVE for protein docking decoy selection, which used a graph neural network (GNN). We used the gate-augmented attention mechanism to capture the atom interaction pattern at the interface region of protein docking models. The benchmark on the Dockground dataset demonstrated that GNN-DOVE outperformed DOVE, along with other existing scoring functions compared. We further trained GNN-DOVE on a larger dataset and evaluated two more datasets, including the CAPRI Score_set, which confirmed superior performance of GNN-DOVE to existing methods.

To assess the quality of structure models, considering multi-body (atom or residue) interactions (Gniewek et al., 2011; Kim and Kihara, 2014; Kim and Kihara, 2016; Olechnovic and Venclovas, 2017) have been proven to be an effective approach. GNNs consider patterns of multiatom interactions by representing the interactions as a graph structure. Since a graph is a natural representation of molecular structures, GNNs may be applied in various problems in structural bioinformatics and cheminformatics.

The performance of GNN-DOVE likely would be improved by considering other physicochemical properties of atoms such as atom-wise binding energies, as well as sequence conservation of residues that can be computed from a multiple sequence alignment of homologous proteins. Application to multichain complexes remains a potential path for future work.

## Data Availability

The datasets presented in this study can be found in online repositories. The Dockground docking dataset was downloaded from the Dockground database (http://dockground.compbio.ku.edu) at the link http://dockground.compbio.ku.edu/downloads/unbound/decoy/decoys1.0.zip. The ZDOCK dataset was downloaded from the ZDOCK decoy sets (https://zlab.umassmed.edu/zdock/decoys.shtml) at the link https://zlab.umassmed.edu/zdock/decoys_bm4_zd3.0.2_6deg.tar.gz. The CAPRI score set was downloaded from http://cb.iri.univ-lille1.fr/Users/lensink/Score_set.
